# Age–Comorbidity Interactions and Clinical Outcomes in Septic Shock: An Emergency Department-Based Multicenter Cohort Study

**DOI:** 10.3390/healthcare14060722

**Published:** 2026-03-12

**Authors:** Seung Jin Maeng, Jong Eun Park, Gun Tak Lee, Sung Yeon Hwang, Minha Kim, Sejin Heo, Tae Ho Lim, Sung Phil Chung, Sung-Hyuk Choi, Tae Gun Shin

**Affiliations:** 1 Department of Emergency Medicine, Samsung Medical Center, Sungkyunkwan University School of Medicine, Seoul 06351, Republic of Korea; sjinmaeng@gmail.com (S.J.M.);; 2 Department of Emergency Medicine, College of Medicine, Hanyang University, Seoul 15495, Republic of Korea; 3 Department of Emergency Medicine, Gangnam Severance Hospital, Yonsei University College of Medicine, Seoul 06273, Republic of Korea; 4 Department of Emergency Medicine, Korea University Guro Hospital, Seoul 08308, Republic of Korea

**Keywords:** sepsis, septic shock, aging, comorbidities, mortality, emergency department

## Abstract

**Highlights:**

**What are the main findings?**

- Age and comorbidities are independently associated with mortality in septic shock.
- There was a significant interaction between age and comorbidity status in mortality.

**What are the implications of the main findings?**

- The strong age–comorbidity interaction suggests that risk assessment should move beyond age alone and incorporate comorbidity profiles in patients with sepsis.

**Abstract:**

**Background**: Sepsis remains a leading cause of mortality worldwide. This study evaluated the independent and combined effects of age and chronic comorbidities on clinical outcomes in patients with septic shock. **Methods**: We conducted a multicenter retrospective observational study to evaluate the factors associated with 28-day mortality in the Korean Shock Society registry between 2015 and 2023. Adults with suspected infection and refractory hypotension or hypoperfusion within 6 h of emergency department (ED) arrival were included. Patients were grouped by age (<50, 50–74, and ≥75 years) and comorbidity status. Comorbidities encompass major chronic conditions including hypertension, diabetes mellitus, malignancy, history of organ transplant, dementia, nursing home residence, chronic disease of cardiac, lung, liver, and kidney. The primary outcome was 28-day mortality. Multivariable logistic regression analysis was used. **Results**: Among 8787 patients (median age 70.2 years), the 28-day mortality rate was 22.9% (n = 2018). Elderly patients with comorbidities had the highest mortality (27.5%). Additionally, patients aged over 50 with at least one comorbidity accounted for 18% of the total cohort (n = 1605) but accounted for nearly 80% of all 28-day deaths. Although younger patients without comorbidities represented a small subgroup, their mortality was not negligible (7.3%) and was substantially higher with comorbidities (22.2%). Compared with patients <50 years, adjusted odds ratios (aORs) of 28-day mortality were 1.81 (95% CI, 1.08–3.03) for 50–74 years and 3.21 (95% CI, 1.92–5.37) for ≥75. The presence of any comorbidities was independently associated with higher odds of 28-day mortality compared with no comorbidity (aOR 2.67; 95% CI, 1.57–4.54). A significant interaction between age and comorbidity status (*p* for interaction = 0.008) suggested that the age-related gradient in mortality differed depending on comorbidity burden. **Conclusions**: Age and comorbidities were both significantly associated with septic shock mortality, and their significant interaction demonstrates effect modification, indicating that the prognostic impact of comorbidities differs by age group and that age-related mortality gradients are influenced by comorbidity burden.

## 1. Introduction

Sepsis is defined as life-threatening organ dysfunction caused by a dysregulated host response to infection, while septic shock represents its most severe manifestation, characterized by profound circulatory, cellular, and metabolic abnormalities requiring vasopressor therapy and elevated lactate levels despite adequate fluid resuscitation [1,2]. In-hospital mortality for septic shock remains unacceptably high, often exceeding 30–50% depending on population and setting [3,4]. Identifying prognostic factors is important for improving sepsis care and tailoring risk-based management [5]. Among the host-related factors, age could be associated with sepsis outcomes [5]. Epidemiologic studies have shown that older patients are affected by sepsis and experience worse clinical outcomes, including increased mortality, prolonged intensive care unit (ICU) stays, and greater likelihood of treatment limitations [6,7,8,9]. Elderly age is often accompanied by immunosenescence, decreased organ function reserve, and higher burden of comorbidities, which collectively contribute to vulnerability during the course of sepsis [9,10]. On the other hand, chronological age alone may not fully capture prognosis, highlighting the need to stratify risk more precisely within age groups [11].

Chronic comorbidities are also determinants of outcomes in sepsis. For instance, conditions such as malignancy, liver cirrhosis, and chronic kidney disease have been independently associated with higher mortality in sepsis patients [12,13,14]. Comorbid conditions could not only increase the likelihood of acquiring sepsis but also impair the host’s ability to recover. The impact of comorbidities on clinical outcomes may be modified by age, and the combined effect of age and comorbid conditions on sepsis outcomes warrants further investigation [6].

Most previous studies have evaluated age and comorbidity burden largely as separate prognostic factors—often adjusting for one while focusing on the other—rather than explicitly examining their joint prognostic effect or interaction on mortality. Moreover, beyond relative risk estimates, the epidemiologic profile of who actually dies—namely the distribution of deaths (“mortality burden”) across age strata and across comorbidity status—remains insufficiently described. Such burden estimates are clinically meaningful because they indicate where most deaths are concentrated and help prioritize prevention, early recognition, and resource allocation strategies.

The objective of this study is to evaluate the independent and combined effects of age and chronic comorbidities on clinical outcomes in patients with septic shock. Utilizing a multicenter registry, we aim to: (1) stratify patients by age groups and comorbidity categories; (2) compare mortality rates across these strata; (3) assess the proportion of total deaths contributed by each group; (4) determine whether age and comorbidities independently affect mortality and have an interaction effect.

## 2. Materials and Methods

### 2.1. Study Design, Setting, Population, Definitions and Outcomes

This retrospective observational study was conducted using a dataset derived from the multicenter Korean Shock Society (KoSS) registry, which prospectively collected data between October 2015 and April 2023 to collect standardized data on septic shock patients seen at the emergency department (EDs) of 12 university-affiliated hospitals. The institutional review board of Samsung Medical Center approved this study. The requirement for informed consent was waived because this study was retrospective, observational, and anonymous. KoSS operates as a nationwide collaborative network aimed at enhancing sepsis diagnosis and treatment strategies. As the implementation of the KoSS registry began before the publication of the Sepsis-3 criteria, the registry inclusion criteria were based on the previous clinical definition of septic shock throughout the study period.

Patients who met all of the following criteria were eligible for inclusion: (1) 18 years of age or older; (2) patients with suspected infection with refractory hypotension or hypoperfusion within 6 h from ED arrival. Refractory hypotension was defined as persistent hypotension, which was systolic blood pressure (SBP) < 90 mm Hg, a mean arterial pressure (MAP) < 70 mm Hg, or an SBP decrease of >40 mm Hg from baseline blood pressure despite intravenous fluid challenge (at least 1 L or 20–30 mL/kg of crystalloid solution administered over 30 min) or as the need for vasopressors after fluid resuscitation. Hypoperfusion was defined as a serum lactate concentration of ≥4 mmol/L [15,16].

Patients were stratified into three age groups: less than 50 years (younger group), 50 to 74 years (middle-aged group), and 75 years or older (elderly group) [17,18]. We defined the presence of comorbidities as having one or more of the following conditions at the time of admission to the ED: hypertension, diabetes mellitus, cardiac disease, chronic lung disease, hematologic malignancy, metastatic cancer, chronic renal disease, chronic liver disease, history of organ transplant, dementia, or a history of residence in a nursing center. Comorbidity was defined to include hematologic or metastatic malignancy, reflecting a higher burden of chronic disease based on prior studies demonstrating that patients with hematologic malignancies and metastatic cancer have higher mortality compared with patients without these conditions. Localized solid tumors without metastasis were not included in this definition [19]. Although nursing home residency is not a traditional comorbidity, we considered it a comorbidity factor because previous studies have shown that residents of nursing homes have higher mortality from severe infections, including sepsis [20,21,22].

Source control interventions included drainage of infected collections, surgical debridement, removal of infected foreign bodies, relief of anatomical obstruction, and surgical decontamination procedures. The Sequential Organ Failure Assessment (SOFA) score was defined as the maximum value recorded within 24 h from ED arrival. The primary outcome was 28-day mortality and the secondary outcome was 90-day mortality.

### 2.2. Primary Data Analysis

Standard descriptive statistics were used for all variables including baseline demographics and outcomes. The results are presented as medians and interquartile ranges (IQRs) for continuous variables and as numbers with percentages for categorical data. Categorical variables were compared using the chi-square test. For continuous variables, comparisons between two independent groups were performed using the Wilcoxon rank-sum test, whereas comparisons among three independent groups were conducted using the Kruskal–Wallis test. When the overall Kruskal–Wallis test was significant, post hoc pairwise comparisons were performed with Bonferroni correction. Univariable and multivariable logistic regression models were developed to assess variables related to 28-day mortality. Effect modification was assessed by including an interaction term between age group and comorbidity status, and statistical significance was evaluated using a Wald test. The following variables were included in the multivariable adjustment model: age, presence of any comorbidities, SOFA score, APACHE II score, infection focus, timely source control (within 12 h), antibiotic administration within 3 h, and serum lactate level. The results were described as adjusted odds ratios (aORs) with 95% confidence intervals (CIs). Survival analyses were performed using the Kaplan–Meier and log-rank methods. A Cox proportional hazards model for 90-day mortality was constructed for the multivariable survival analyses adjusting the selected variables. A *p*-value less than 0.05 was considered to indicate a statistically significant difference. STATA 15.0 (StataCorp LLC, College Station, TX, USA) was used for statistical analysis.

## 3. Results

### 3.1. Baseline Characteristics

**Figure 1:**
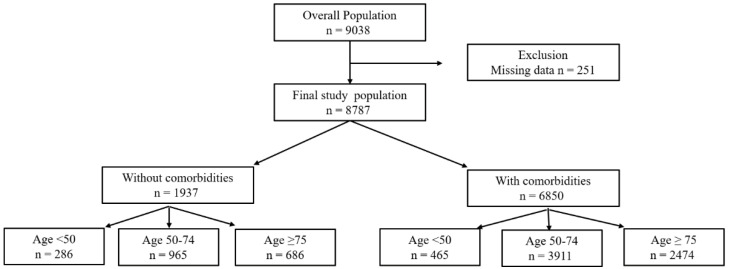
Study population.

A total of 8787 patients were included from the registry (Figure 1). The median age was 70.2 (IQR, 61.0–78.6) years, 57.3% were male, and the overall 28-day mortality rate was 22.9% (2018 deaths). The overall 90-day mortality rate was 34.1% (2996 deaths, 639 missing data). Intra-abdominal infection was the most common source of infection (31.4%), and the median SOFA score was 8 (IQR, 6–11). Patients who died within 28 days were significantly older (median age 71.7 [IQR, 62.4–80.3] vs. 69.8 [IQR, 60.6–78.1] years) and more frequently had underlying comorbidities (84.6% vs. 76.0%) than those who survived (*p* < 0.001 for both comparisons; Table S1).

Age groups were defined as <50, 50–74, and ≥75 years; comorbidity was defined as the presence of at least one predefined chronic condition.

Patient characteristics according to age strata are described in Table 1. The cohort consisted of 751 (8.6%) patients aged < 50 years, 4876 (55.5%) aged 50–74 years, and 3160 (36.0%) aged ≥ 75 years. Respiratory infection was more common in the elderly group at 26.4% compared to other age groups. The rates of mechanical ventilation (30.3%) and ICU admission (59.9%) were also highest in the elderly group.

Patient characteristics according to the presence or absence of comorbidities are presented in Table S2. Of the 8787 patients, 6850 (78.0%) had at least one. While patients with comorbidities had higher median SOFA scores (8 vs. 7), higher lactate levels (3.6 vs. 3.1 mmol/L), and a greater need for renal replacement therapy (15.7% vs. 12.0%), their rate of ICU admission was slightly lower (57.1% vs. 60.9%). While respiratory infections were more frequent among patients with comorbidities (24.2% vs. 19.8%), intra-abdominal infections were more common in those without comorbidities (34.4% vs. 30.5%).

### 3.2. Outcomes

**Figure 2:**
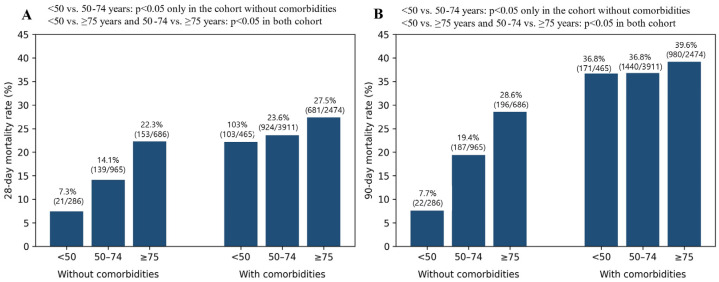
28-day (**A**) and 90-day (**B**) mortality stratified by age and comorbidity status.

Overall, 2018 of 8787 patients (22.97%) died within 28 days. Crude 28-day mortality increased across age groups, from 16.5% (124/751) in patients aged < 50 years to 21.7% (1060/4876) in those aged 50–74 years and 26.4% (834/3160) in those aged ≥ 75 years. Mortality was also higher in patients with comorbidities than in those without (24.9% vs. 16.0%, *p* < 0.001). In the cohort of patients without comorbidities, pairwise comparisons showed a significant increase in both 28-day and 90-day mortality between <50 years and 50–74 age groups, as well as between the 50–74 and ≥75 age groups (*p* < 0.05 for all comparisons). In contrast, among patients with comorbidities, the age-related increase in mortality was less pronounced, and no statistically significant difference was observed in 28-day and 90-day mortality between the <50 and 50–74 years groups, whereas mortality was significantly higher in patients aged ≥ 75 years compared with younger age groups (Figure 2).

Age groups were defined as <50, 50–74, and ≥75 years; comorbidity was defined as the presence of at least one predefined chronic condition.

The 28- and 90-day mortality differences between the <50 and 50–74 age groups were significant only in the cohort without comorbidities (*p* < 0.05). In contrast, differences between <50 and ≥75, and between 50–74 and ≥75, were significant regardless of comorbidity status (*p* < 0.05). Accordingly, age-related mortality differences between age groups were observed primarily among patients without comorbidities.

**Figure 3:**
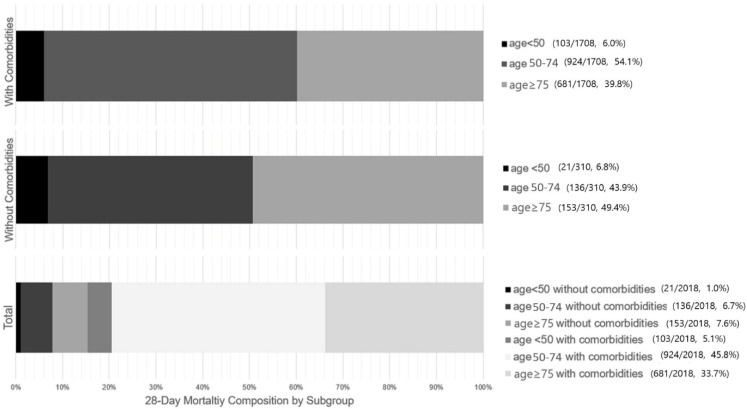
Proportional contribution of age and comorbidity subgroups to 28-day mortality.

Figure 3 illustrates the proportional distribution of all 28-day deaths across the subgroups, highlighting the mortality burden. Among patients who experienced 28-day mortality (n = 2018, 22.9% of the overall cohort), patients aged ≥ 50 years with comorbidities accounted for nearly 80% of all deaths (79.5%), as illustrated in Figure 3. In contrast, the contribution from non-elderly patients (<75 years) without comorbidities was less than 10%. (Figure 3, Total panel).

Age groups were defined as <50, 50–74, and ≥75 years; comorbidity was defined as the presence of at least one predefined chronic condition. Absolute numbers (deaths/total patients) and corresponding percentages for each subgroup are shown alongside each panel.

The figure illustrates the distribution of 28-day mortality stratified by age groups, shown separately for patients with comorbidities (top panel), without comorbidities (middle panel), and the total 28-day mortality population (bottom panel). Of those who died within 28 days, 79.5% were aged > 50 with at least one comorbidity, while 7.7% were aged < 75 and had no comorbidities. Accordingly, a measurable proportion of 28-day death occurred in patients without advanced age or comorbidity.

### 3.3. Regression Analyses

The results from univariable logistic regression to identify the risk factors for 28-day mortality are presented in Table S3, and those from multivariable analysis are presented in Table 2. Compared to patients younger than 50, the aOR for 28-day mortality was 1.81 (95% CI, 1.08–3.03) for the 50–74 years group and increased to 3.21 (95% CI, 1.92–5.37) for the ≥75 years group. The presence of any comorbidity was also associated with 28-day mortality (aOR 2.67; 95% CI, 1.57–4.54) (Table 2). Importantly, a significant interaction was observed between age and comorbidity status (*p* for interaction = 0.008). To clarify the significant interaction, Table S4 presents age-stratified adjusted odds ratios for the presence of comorbidity using a multivariable logistic regression model. Younger patients (<50 years) with comorbidities showed a significantly increased adjusted odds of 28-day mortality (aOR, 2.67; 95% CI, 1.57–4.54) when the patients aged < 50 years without comorbidities were used as the reference group (Table S4). Also, older age was associated with progressively higher adjusted odds of 28-day mortality with an aOR of 2.98 in those aged 50–74 years and 3.86 in those aged ≥ 75 years, explaining the significant age–comorbidity interaction (Table S4). A margins plot illustrating the adjusted probability of 28-day mortality across age and comorbidity subgroups showed a graded increase in mortality risk consistent with the adjusted odds ratios (Figure S1). Table S4 and Figure S1 demonstrate that, in the absence of comorbidities, mortality risk increased progressively with age, whereas in the presence of comorbidities, elevated mortality risk was observed across all age groups, including younger patients.

### 3.4. Survival Analyses

**Figure 4:**
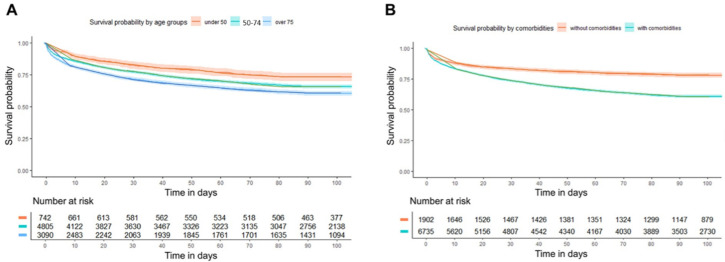
Kaplan–Meier curves for 90-day survival according to (**A**) age strata and (**B**) comorbidities.

Kaplan–Meier curves demonstrated significantly reduced survival in patients aged ≥ 75 compared to younger cohorts (log-rank *p* < 0.001) (Figure 4A). Similarly, patients with comorbidities had markedly lower survival rates than those without (log-rank *p* < 0.001) (Figure 4B). For 90-day mortality, a Cox proportional hazards model was used. Compared to patients younger than 50, the adjusted hazard ratio (aHR) for 90-day mortality was 1.20 (95% CI, 1.04–1.40) for the 50–74 years group and 1.57 (95% CI, 1.35–1.83) for the ≥75 years group. The presence of comorbidities was associated with an aHR of 1.56 (95% CI, 1.41–1.74) (Table S5).

Age groups were defined as <50, 50–74, and ≥75 years; comorbidity was defined as the presence of at least one predefined chronic condition.

Survival rates significantly differed among age groups (log-rank test, *p* < 0.001 for all pairwise comparisons). Survival rates also significantly differed between patients with and without comorbidities (log-rank test, *p* < 0.001). These survival patterns demonstrate distinct survival differences according to age group and comorbidity status.

## 4. Discussion

Although several recent studies report that sepsis-related mortality has been slowly declining, it nonetheless remains high. Meanwhile, the incidence of sepsis appears to be increasing—a trend that may be partially explained by population aging and increasing prevalence of chronic comorbid conditions [12,23,24]. To inform risk stratification at initial ED presentation, we analyzed a large, multicenter, ED-based registry of patients with septic shock. We evaluated the independent and interactive effects of age and comorbidity on short- and mid-term mortality. By including not only elderly patients and those with chronic diseases but also younger adults without comorbidities, we characterized the epidemiologic profile of septic shock across the full adult age spectrum. Furthermore, we estimated the contribution of each age–comorbidity subgroup to the overall mortality burden (i.e., the distribution of total deaths across strata). This provides a population-level perspective on where deaths are concentrated and estimates the proportion of septic shock deaths occurring in patients without advanced age or chronic comorbidities—a subgroup that may represent a more potentially preventable fraction of sepsis-associated mortality.

The finding that advanced age is significantly associated with mortality in patients with sepsis is consistent with the results of previous epidemiologic studies [23,25,26]. While aging was previously regarded simply as a chronological process, the influence of age on sepsis prognosis arises from complex mechanisms. Firstly, aging leads to a gradual decline in organ system reserve, impairing the body’s ability to cope with acute physiological stress [27]. Secondly, older patients may experience impaired immune responses, a concept known as immunosenescence, which can hinder an appropriate reaction to infection and potentially lead to higher mortality [28]. Thirdly, the increasing prevalence of chronic comorbid conditions with advancing age was strongly associated with greater sepsis severity and higher risk of mortality [12].

Yet, there is no universally accepted age stratification to sepsis. In sepsis-related studies, various age cut-offs have been frequently adopted. In this study, we classified age as <50, 50–74, and ≥75 years based on prior sepsis literature [17,18].

On the other hand, interpreting age as a single risk factor has its limitations. One study found that age was an independent risk factor for death only in the “very elderly” (≥80 years) patient group [29], while another demonstrated that younger patients with comorbidities could be at higher risk than older adults without them, suggesting the burden of comorbidity can sometimes have a greater impact on prognosis than age itself [27]. In our study, while we observed that an age of ≥75 years tripled the adjusted 28-day mortality risk relative to those <50 years, this effect was most pronounced in patients without chronic diseases and was attenuated in the cohort already burdened by comorbidities. It is intriguing that most patients with comorbidities fell into the 50–74 years group rather than the elderly group. This may be related to the underlying population structure of our cohort. In a tertiary hospital setting, patients with malignancy and other serious chronic conditions are more prevalent than in the general population. Because of this, when comparing 28-day mortality, the 50–74 years group with comorbidities represented the largest proportion of deaths.

Outcomes in young and healthy populations are relatively under-investigated. Prior studies have reported that mortality among young patients with sepsis ranged from 5.8% to 24%. The results of Alrawashdeh et al. (5.8%) and Lavioud et al. (11.7%) were similar to those of our study [30,31,32]. Although our study showed that most deaths occurred in older patients or those with chronic comorbidities, it also revealed that mortality in the younger cohort (<50 years) was not negligible (22.2% with comorbidities, 7.3% without). These findings indicate that the presence of comorbidities substantially elevates mortality risk even among younger patients, resulting in a risk profile comparable to that of older age groups and supporting the observed age–comorbidity interaction. The relative impact of comorbidities on mortality appeared more pronounced in younger patients, in whom baseline mortality risk is low without comorbidity, whereas age-related baseline vulnerability may attenuate the effect of comorbidity in older patients. Accordingly, sepsis severity in younger patients should not be underestimated, particularly when significant comorbidities are present. These findings suggest that age alone may be insufficient for prognostic assessment and that age and comorbidity burden should be considered jointly in clinical risk stratification.

Beyond the age-specific findings described above, chronic health conditions are highly prevalent in the septic population and have been identified as independent risk factors that aggravate disease severity and increase the risk of death [24,33,34]. In older adults, this risk is compounded, as the higher prevalence of comorbidities converges with an age-related decline in physiological reserve, creating a state of heightened vulnerability to sepsis [12,27]. Sepsis is frequently the direct cause of death in hospitalized patients; however, underlying chronic health conditions play a major role in determining sepsis-associated mortality [24]. Thomas-Ruddel et al. [35] also reported that deaths in elderly patients with comorbidities are often attributable to their underlying conditions rather than sepsis alone. Consistent with previous studies, our study revealed that nearly 80% of ED patients with septic shock had at least one chronic comorbidity with higher mortality. These results, along with previous studies, suggest that reducing sepsis mortality requires not only optimal acute sepsis treatment but also improved prevention and management of the underlying diseases that create vulnerability to sepsis.

This study has several important limitations. First, although we adjusted for key markers of acute illness severity, residual and unmeasured confounding cannot be excluded. Second, we could only record the presence or absence of predefined chronic conditions; therefore, neither the full spectrum nor the quantitative burden (e.g., Charlson Comorbidity Index, frailty score) of underlying diseases was assessed. Our definition of comorbidity did not incorporate cumulative indices such as the Charlson Comorbidity Index or frailty scores, which were unavailable in our registry and are primarily designed for long-term mortality prediction. Instead, this study focused on age and comorbidity information readily available at ED triage, supporting practical short-term risk stratification. Third, to avoid excluding earlier patients and maintain consistency across the entire study period, we used the original KoSS septic shock definition applied prospectively at the initiation of the registry. Therefore, we inevitably had to rely on a definition that predated the Sepsis-3 Consensus criteria. In addition, the registry is confined to EDs in academic hospitals, and the data were collected during the COVID-19 pandemic. These factors may limit generalizability of our findings, particularly when applied to other healthcare systems or non-ED settings in the post-pandemic era. Due to the observational nature of our study, causal relationships cannot be established. Lastly, given the large sample size of this registry, some statistically significant findings may not necessarily reflect clinically meaningful differences.

Although the KoSS registry predates Sepsis-3, the median SOFA score was 8 (IQR, 6–11), indicating disease severity comparable to Sepsis-3 cohorts, and a substantial proportion of patients met the Sepsis-3 criteria (5874 [66.9%]), supporting the applicability of our findings to contemporary ED settings.

## 5. Conclusions

Age and the presence of comorbidities were significantly associated with mortality in patients with septic shock presenting to the EDs.

A significant interaction between age and comorbidity was observed, indicating that their prognostic effects are interdependent. Clinically, age and comorbidity should be considered jointly when assessing prognosis in patients with septic shock. Although identified in a multicenter ED cohort, further studies are needed to confirm the generalizability.

## Figures and Tables

**Table 1:** Baseline characteristics according to age strata.

Total n = 8787	Age < 50 n = 751 (8.6%)	Age 50–74 n = 4876 (55.5%)	Age ≥ 75 n = 3160 (36.0%)	*p*-Value
**Male (n, %)**	390 (51.9) ^||^	2992 (61.4) ^¶^	1652 (52.3)	<0.001
**Infection focus (n, %)**	^||^	^¶^	^#^	
Respiratory	116 (15.5)	1089 (22.3)	834 (26.4)	<0.001
Urinary tract	129 (17.2)	752 (15.4)	700 (22.2)	<0.001
Intra-abdominal	222 (29.6)	1719 (35.3)	814 (25.8)	<0.001
Others	284 (37.8)	1316 (27.0)	812 (25.7)	<0.001
**Comorbidities (n, %)**				
Hypertension	93 (12.4)	1806 (37.0)	1845 (58.4)	<0.001
Diabetes	118 (15.7) ^||^	1.552 (31.8) ^¶^	1189 (37.6) ^#^	<0.001
Chronic cardiac disease	23 (3.1) ^||^	528 (10.8) ^¶^	705 (22.3) ^#^	<0.001
Chronic lung disease	11 (1.5) ^||^	333 (6.8) ^¶^	336 (10.6) ^#^	<0.001
Hematologic malignancy	88 (11.7) ^||^	439 (9.0) ^¶^	128 (4.1) ^#^	<0.001
Metastatic cancer	194 (25.8) ^||^	1732 (35.5) ^¶^	580 (18.4) ^#^	<0.001
Chronic renal disease	59 (7.8)	407 (8.4) ^¶^	350 (11.1) ^#^	<0.001
Chronic liver disease	85 (11.3)	639 (13.1) ^¶^	169 (5.4) ^#^	<0.001
Solid organ transplantation	42 (5.6) ^||^	178 (3.7) ^¶^	13 (0.4) ^#^	<0.001
Dementia	4 (0.5)	101 (2.1) ^¶^	449 (14.2) ^#^	<0.001
Nursing Center	31 (4.1)	331 (6.8) ^¶^	516 (16.3) ^#^	<0.001
**Any comorbidity (n, %)**	465 (61.9) ^||^	3911 (80.2)	2474 (78.3) ^#^	<0.001
**Initial Vital Signs (median, IQR)**				
SBP (mmHg)	90 (77–109)	92 (78–112) ^¶^	96 (80–120) ^#^	<0.001
DBP (mmHg)	56 (47–68)	57 (49–68)	56 (47–70)	0.133
HR (bpm)	120 (102–136) ^||^	111 (95–128) ^¶^	104 (88–120) ^#^	<0.001
RR (bpm)	20 (18–22) ^||^	20 (18–24) ^¶^	20 (18–25) ^#^	<0.001
BT (°C)	37.9 (36.9–38.9)	37.6 (36.7–38.6) ^¶^	37.4 (36.5–38.4) ^#^	<0.001
**SOFA score (median, IQR)**	8 (5–11) ^||^	8 (6–11)	8 (6–11)	0.017
**New definition of Sepsis-3**				
**Septic shock** 5874 (66.9%)	465 (61.9) ^||^	3275 (67.2)	2134 (67.5) ^#^	0.010
**Laboratory (median, IQR)**				
WBC (×10^3^/μL)	9.1 (3.5–16.4)	8.9 (3.5–15.9) ^¶^	11.9 (6.8–18.0) ^#^	<0.001
Creatinine (mg/dL)	1.2 (0.8–2.2)	1.3 (0.9–2.1)	1.5 (1.0–2.3)	0.105
Albumin (mg/dL)	3.1 (2.5–3.7) ^||^	2.9 (2.5–3.4)	3.0 (2.5–3.4) ^#^	<0.001
PT (INR)	1.3 (1.1–1.5)	1.3 (1.1–1.5)	1.2 (1.1–1.4)	0.071
CRP (mg/dL)	12.3 (3.9–23.7)	13.9 (5.9–24.1)	13.6 (5.6–22.2)	0.080
Lactate (mmol/L)	3.0 (1.7–5.2) ^||^	3.4 (1.9–5.6) ^¶^	2.1 (3.7–5.9) ^#^	<0.001
**Sepsis Treatments** *				
Vasopressor use ^†^ (n, %)	659 (87.8)	4312 (88.4) ^¶^	2703 (85.5)	0.001
Time to vasopressor (hours, median, IQR) ^‡^	2.6 (1.5–4.4)	2.4 (1.2–4.2)	2.3 (1.2–4.1)	0.485
Time to interventions for source control in hours (median, IQR) ^†^	19.2 (8.0–56.7)	15.4 (7.6–42.2) ^¶^	12.4 (6.7–26.1)	0.014
Time to antibiotic therapy in hours (median, IQR) ^§^	2.6 (1.5–3.9) ^||^	2.3 (1.4–3.7)	2.3 (1.4–3.6) ^#^	0.028
RRT (n, %)	119 (15.8)	732 (15.0)	458 (14.5)	0.610
MV (n, %)	182 (24.2) ^||^	1384 (28.4)	956 (30.3) ^#^	0.004
ICU admission (n, %)	420 (55.9)	2775 (56.9) ^¶^	1895 (59.9) ^#^	0.013

Abbreviations: IQR, interquartile range; SBP, systolic blood pressure; DBP, diastolic blood pressure; HR, heart rate; bpm, beats per minutes; RR, respiratory rate; BT, body temperature; SOFA: maximum Sequential Organ Failure Assessment score within 24 h from Emergency Department (ED) arrival; WBC, white blood cell; PT, prothrombin time; INR, international normalized ratio; CRP, C-reactive protein, RRT, renal replacement therapy; MV, mechanical ventilation; ICU, intensive care unit. * Sepsis Treatments include vasopressor use, interventions, antibiotics use, adjunctive organ support such as RRT, MV, and ICU admission. ^†^ Vasopressor use and Interventions were performed within 24 h after emergency department arrival. ^‡^ Vasopressor duration was calculated by subtracting the ending time when the vasopressor was not in use over a continuous 6 h from the first administration time. ^§^ The time to antibiotic therapy was calculated by subtracting the emergency department triage time from the first time of broad-spectrum antibiotic administration. ^||^ A statistically significant difference was observed between the age < 50 group and the age 50–74 group. ^¶^ A statistically significant difference was observed between the age 50–74 and the age ≥ 75 group. ^#^ A statistically significant difference was observed between the age < 50 and the age ≥ 75 group. ^||^, ^¶^, ^#^ indicate significant difference compared with the <50-year group after Bonferroni correction.

**Table 2:** Multivariable logistic regression analysis for age strata and comorbidities for 28-day mortality.

	28-Day Mortality		
	aOR *	95% CI	*p*-Value
Age < 50	Reference		
Age 50–74	1.71	(1.02–2.87)	0.042
Age ≥ 75	3.07	(1.83–5.16)	<0.001
Comorbidities	2.50	(1.46–4.26)	0.001
SOFA score	1.15	(1.13–1.17)	<0.001
APACHE II	1.02	(1.01–1.03)	<0.001
Infection focus			
Respiratory	Reference		
Urinary tract	0.33	(0.27–0.40)	<0.001
Intra-abdominal	0.56	(0.48–0.65)	<0.001
Others	0.67	(0.58–0.77)	<0.001
Source control ^†^	0.52	(0.42–0.65)	<0.001
Antibiotics use ^‡^	0.83	(0.74–0.93)	0.002
Lactate	1.16	(1.14–1.17)	<0.001

Abbreviations: CI, confidence interval; aOR, adjusted odds ratio; SOFA, maximum Sequential Organ Failure Assessment score within 24 h from Emergency Department (ED) arrival. * Adjusted variables were age, comorbidities, maximum SOFA, Lactate, APACHEII (Acute Physiology and chronic health evaluation II), Infection focus, Source control within 12 h, Administration of antibiotics within 3 h after ED arrival. ^†^ Source control when performed within 12 h after ED arrival. ^‡^ Administration of antibiotics within 3 h after ED arrival. Note: Interaction between age groups and comorbidity was statistically significant (*p* for interaction = 0.008).

## Data Availability

The datasets used and/or analyzed during the current study are available from the corresponding author on reasonable request.

## Supplementary Materials

The following supporting information can be downloaded at https://www.mdpi.com/article/10.3390/healthcare14060722/s1. Table S1. Baseline characteristics according to survival. Table S2. Baseline characteristics according to comorbidities. Table S3. Univariable logistic regression analysis for age strata and comorbidities for 28-day mortality. Table S4. Adjusted odds ratios for 28-day mortality according to age group and presence of any comorbidities. Table S5. Univariable and multivariable hazard ratios for 90-day mortality. Figure S1. Adjusted probability of 28-day mortality by age group and presence of any comorbidities.
