# Supplementary material for: Age–Comorbidity Interactions and Clinical Outcomes in Septic Shock: An Emergency Department-Based Multicenter Cohort Study
Source: Healthcare (Basel). 2026 Mar 12;14(6):722. doi: 10.3390/healthcare14060722 (PMC13026241; doi:10.3390/healthcare14060722)
Supplement: Supplementary file 1 [file healthcare-14-00722-s001.zip › healthcare-4071432-supplementary.pdf]

Supplementary Materials

Table S1. Baseline characteristics according to survival

	Total (n=8,787)	Survival (n=6,769)	28-day Death (n=2,018)	p-value
**Age (median, IQR)**	70.2 (61.0-78.6)	69.8 (60.6-78.1)	71.7 (62.4-80.3)	<0.001
**Age strata (n, %)**				<0.001
<50	751 (8.6)	627 (9.3)	124 (6.1)	
50-75	4,876 (55.5)	3,816 (56.4)	1,060 (52.5)	
≥75	3,160 (35.9)	2,326 (34.3)	834 (41.3)	
**Male (n, %)**	5,034 (57.3)	3,792 (56.0)	2,977 (44.0)	<0.001
**Comorbidities (n, %)**				
Hypertension	3744 (42.6)	2,876 (42.5)	868 (43.0)	0.675
Diabetes mellitus	2,859 (32.5)	2,188 (32.3)	671 (33.3)	0.435
Chronic cardiac disease	1,256 (14.3)	944 (14.0)	312 (15.5)	0.088
Chronic lung disease	680 (7.7)	476 (7.0)	204 (10.1)	<0.001
Hematologic malignancy	655 (7.5)	458 (6.8)	197 (9.8)	<0.001
Metastatic cancer	2,506 (28.5)	1,772 (26.2)	734 (36.4)	<0.001
Chronic renal disease	798 (9.1)	598 (8.8)	200 (9.9)	0.140
Chronic liver disease	893 (10.2)	649 (9.6)	244 (12.1)	0.001
Transplant	233 (2.7)	211 (3.1)	22 (1.1)	<0.001
Dementia	554 (6.3)	394 (5.8)	160 (7.9)	0.001
Nursing home residence	878 (10.0)	626 (9.3)	252 (12.5)	<0.001
Composite	6,850 (78.0)	5,142 (76.0)	1,708 (84.6)	<0.001
**Focus (n, %)**				<0.001
Respiratory	2,039 (23.2)	1,351 (20.0)	688 (34.1)	
Urinary tract	1,581 (18.2)	1,392 (20.6)	189 (9.4)	
Intra-abdominal	2,755 (31.4)	2,225 (32.9)	530 (26.3)	
Others	2,412 (27.5)	1,801 (26.6)	611 (30.3)	
**Vital Signs (median, IQR)**				
SBP, mmHg	93 (79-115)	93 (79-115)	92 (77-116)	0.098
DBP, mmHg	57 (48-69)	57 (49-68)	56 (47-70)	0.059
HR, bpm	110 (92-126)	109 (92-125)	112 (96-129)	<0.001
RR, bpm	20 (18-24)	20 (18-24)	22 (20-28)	<0.001
BT, °C	37.6 (36.6-38.6)	37.8 (36.8-38.7)	37 (36.3-38)	<0.001
**SOFA (median, IQR)**	8 (6-11)	8 (5-10)	11 (8-14)	<0.001
**New definition of Sepsis-3**				
**Septic shock**	5874 (66.9)	4261 (63.0)	1613 (79.9)	<0.001
**Laboratory (median, IQR)**				
WBC($x10^3/\mu L$)	10.2 (4.6-16.8)	10.4 (5.1-16.7)	9.2 (2.8-17.4)	<0.001
Creatinine (mg/dL)	1.4 (0.9-2.2)	1.3 (0.9-2.1)	1.6 (1.1-2.6)	<0.001
Albumin (mg/dL)	2.9 (2.5-3.4)	3 (2.6-3.5)	2.6 (2.2-3.1)	<0.001
PT (INR)	1.3 (1.1-1.5)	1.2 (1.1-1.4)	1.4 (1.2-1.7)	<0.001
CRP (mg/dL)	13.6 (5.6-23.4)	13.0 (5.2-22.6)	15.9 (7.2-25.8)	<0.001
Lactate (mmol/L)	3.4 (2.0-5.6)	3.1 (1.8-4.9)	5.1 (2.9-8.2)	<0.001

Abbreviations: IQR, interquartile range; SBP, systolic blood pressure; DBP, diastolic blood pressure; MAP, mean arterial pressure; HR, heart rate; RR, respiratory rate; BT, body temperature; SOFA, maximum Sequential Organ Failure Assessment score within 24 hours of arrival to the emergency department; WBC, white blood cell; PT, prothrombin time; INR, international normalized ratio; CRP, C-reactive protein.

Table S2. Baseline characteristics according to comorbidities

	Without comorbidities (n=1,937, 22%)	With comorbidities (n=6,850/8787, 78%)	*p*-value
**Age, years (median, IQR)**	70.3 (61.6-78.6)	69.7 (57.9-78.7)	<0.001
**Age strata (n, %)**			<0.001
<50 years	286 (14.8)	465 (6.8)	
50-74 years	965 (49.8)	3,911 (57.1)	
≥75 years	686 (35.4)	2,474 (36.1)	
**Male (n, %)**	1,026 (53.0)	4,008 (58.5)	<0.001
**Focus (n, %)**			<0.001
Respiratory	384 (19.8)	1,655 (24.2)	
Urinary tract	384 (19.8)	1,197 (17.5)	
Intra-abdominal	667 (34.4)	2,088 (30.5)	
Others	502 (25.9)	1,910 (27.9)	
**Vital Signs (median, IQR)**			
SBP, mmHg	94 (80-116)	93 (78-115)	0.250
DBP, mmHg	57 (49-70)	57 (48-69)	0.151
HR, bpm	107 (91-123)	110 (93-127)	<0.001
RR, bpm	20 (18-24)	20 (18-24)	0.054
BT, °C	37.7 (36.7-38.7)	37.6 (36.6-38.6)	0.001
**SOFA score (median, IQR)**	7 (5-10)	8 (6-11)	<0.001
**New definition of Sepsis-3**			
**Septic shock**	1214 (62.7)	4660 (68.0)	<0.001
**Laboratory (median, IQR)**			
WBC(x10^3/μL)	11.4 (5.9-17.4)	9.9 (4.2-16.6)	<0.001
Creatinine (mg/dL)	1.4 (0.9-2.2)	1.3 (0.9-2.0)	<0.001
Albumin (mg/dL)	3.1 (2.7-3.6)	2.9 (2.4-3.4)	<0.001
PT (INR)	1.2 (1.1-1.4)	1.3 (1.1-1.5)	<0.001
CRP (mg/dL)	14.5 (13.4-24.4)	13.4 (5.7-23.1)	0.498
Lactate (mmol/L)	3.1 (1.7-5.1)	3.6 (2.0-5.8)	<0.001
**Sepsis Treatments**[*]			
Vasopressor use[†] (n, %)	1,669 (86.2)	6,005 (87.7)	0.080
Vasopressor duration in hours (median, IQR) [‡]	2.5 (1.3-4.2)	2.4 (1.2-4.2)	0.117
Time to interventions for source control in hours (median, IQR) [†]	13.5 (7.0-26.6)	15.1 (7.5-42.1)	0.011
Time to antibiotic therapy in hours (median, IQR) [§]	2.4 (1.5-3.8)	2.3 (1.4-3.6)	<0.001
RRT (n, %)	233 (12.0)	1,076 (15.7)	<0.001
MV (n, %)	550 (28.4)	2,002 (29.2)	0.494
ICU admission (n, %)	1,180 (60.9)	3,910 (57.1)	0.003

Abbreviations: IQR, interquartile range; SBP, systolic blood pressure; DBP, diastolic blood pressure; MAP, mean arterial pressure; HR, heart rate; RR, respiratory rate; BT, body temperature; SOFA,: maximum Sequential Organ Failure Assessment score within 24 hours from Emergency Department (ED) arrival ; WBC, white blood cell; PT, prothrombin time; INR, international normalized ratio; CRP, c-reactive protein; RRT, renal replacement therapy; MV, mechanical ventilation; ICU, intensive care unit.

[*]Sepsis Treatments include vasopressor use, interventions, antibiotics use, performing blood cultures.

[†] Vasopressor use and Interventions were performed within 24 hours after emergency department arrival.

[‡] Vasopressor duration was calculated by subtracting the ending time when vasopressor was not in-use over continuous 6hours from the first administration time.

[§] The time to antibiotic therapy was calculated by subtracting the emergency department triage time from the first time of broad-spectrum antibiotics administration.

Table S3. Univariable logistic regression analysis for age strata and comorbidities for 28-day mortality

	28-day mortality		
	OR	95% CI	p-value
**Age <50**	Reference		
Age 50-75	1.41	(1.15-1.73)	<0.001
Age ≥75	1.81	(1.47-2.23)	<0.001
**With comorbidities**	1.74	(1.53-1.99)	<0.001
Diabetes mellitus	1.04	(0.94-1.16)	0.436
Chronic cardiac disease	1.13	(0.98-1.30)	0.090
Chronic lung disease	1.49	(1.25-1.77)	<0.001
Hematologic malignancy	1.49	(1.25-1.78)	<0.001
Metastatic cancer	1.61	(1.45-1.79)	<0.001
Chronic renal disease	1.15	(0.98-1.37)	0.090
Chronic liver disease	1.30	(1.11-1.51)	0.001
Transplant	0.34	(0.22-0.53)	<0.001
Dementia	1.39	(1.15-1.68)	0.009
Nursing home residence	1.40	(1.19-1.64)	<0.001
**SOFA**	1.24	(1.22-1.26)	<0.001
**Lactate**	1.21	(1.19-1.23)	<0.001

Abbreviations: OR, odds ratio; CI, confidence interval; CRP, C-reactive protein; SOFA, maximum Sequential Organ Failure Assessment score within 24 hours of arrival to the emergency department.

Table S4. Adjusted odds ratios for 28-day mortality according to age group and presence of any comorbidities

Age group	Comorbidity	Adjusted odds ratio	95% CI	p-value
<50 years	No	Reference	–	–
<50 years	Yes	2.67	(1.57–4.54)	<0.001
50–74 years	No	1.81	(1.08–3.03)	0.023
50–74 years	Yes	2.98	(1.84–4.83)	<0.001
≥75 years	No	3.21	(1.92–5.37)	<0.001
≥75 years	Yes	3.86	(2.38–6.27)	<0.001

Abbreviations: CI, confidence interval; SOFA, maximum Sequential Organ Failure Assessment score within 24 hours from Emergency Department (ED) arrival.

Adjusted variables were age, comorbidities, maximum SOFA, and Lactate.

Patients aged <50 years without comorbidities served as the reference group.

Table S5. Univariable and multivariable hazard ratios for 90-day mortality

	Univariable		Multivariable	
Parameter	HR (95% CI)	p-value	HR (95% CI)	p-value
**Age** <50	Reference			
Age 50-75	1.33 (1.15-1.55)	<0.001	1.20 (1.04-1.40)	0.015
Age ≥ 75	1.70 (1.46-1.98)	<0.001	1.57 (1.35-1.83)	<0.001
**Comorbidities**	1.83 (1.65-2.03)	<0.001	1.56 (1.41-1.74)	<0.001
**SOFA**	1.17 (1.15-1.18)	<0.001	1.13 (1.12-1.14)	<0.001
**Lactate**	1.13 (1.12-1.14)	<0.001	1.10 (1.09-1.11)	<0.001

Abbreviation: HR, hazard ratio; CI, confidence interval; SOFA, maximum Sequential Organ Failure Assessment score within 24 hours of arrival to the emergency department

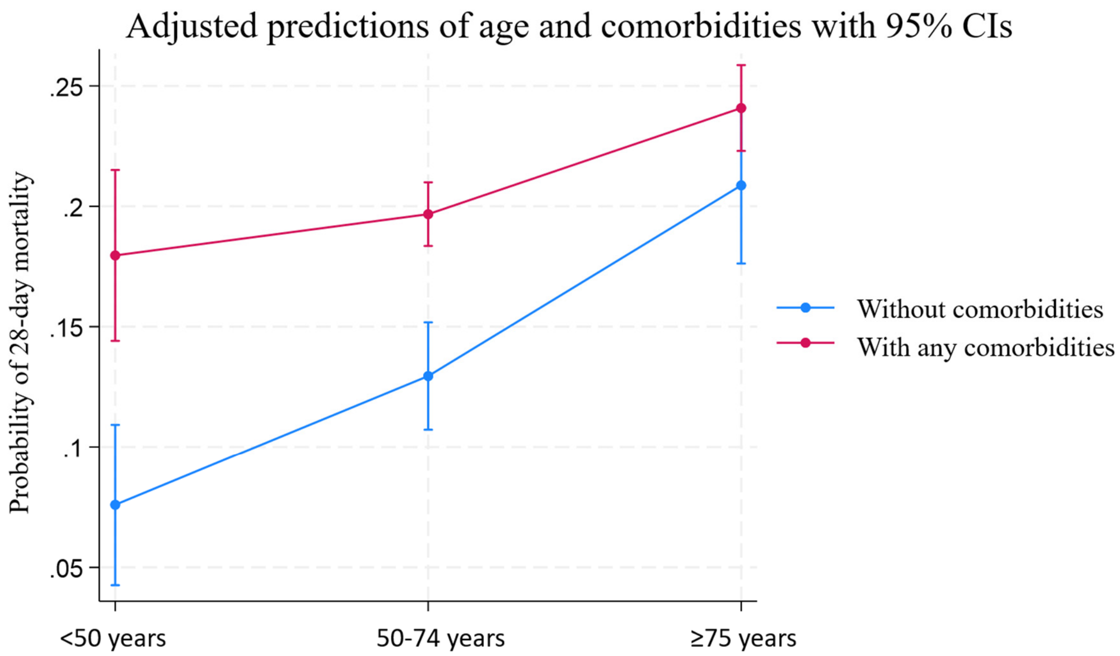

Figure S1. Adjusted probability of 28-day mortality by age group and presence of any comorbidities

Age groups were defined as <50, 50-74, and ≥75 years; and comorbidity was defined as the presence of at least one predefined chronic condition.

The figure illustrates 28-day mortality probabilities with 95% confidence intervals across age and comorbidity subgroups. The contrast in mortality between patients with and without comorbidities appeared more pronounced in younger age group (<50 years) than in older age group (≥75 years).

Abbreviations: CI, confidence interval
